# Targeted disruption of TC-PTP in the proliferative compartment augments STAT3 and AKT signaling and skin tumor development

**DOI:** 10.1038/srep45077

**Published:** 2017-03-21

**Authors:** Hyunseung Lee, Mihwa Kim, Minwoo Baek, Liza D. Morales, Ik-Soon Jang, Thomas J. Slaga, John DiGiovanni, Dae Joon Kim

**Affiliations:** 1 Department of Biomedical Sciences, School of Medicine, University of Texas Rio Grande Valley, Edinburg, TX 78541, USA; 2 Division of Bioconvergence Analysis, Korea Basic Science Institute, Daejeon 305-333, Republic of Korea; 3 Department of Pharmacology, School of Medicine, University of Texas Health Science Center at San Antonio, San Antonio, TX 78229, USA; 4 Division of Pharmacology & Toxicology, College of Pharmacy, The University of Texas at Austin, Austin, TX 78723, USA.

## Abstract

Tyrosine phosphorylation is a vital mechanism that contributes to skin carcinogenesis. It is regulated by the counter-activities of protein tyrosine kinases (PTKs) and protein tyrosine phosphatases (PTPs). Here, we report the critical role of T-cell protein tyrosine phosphatase (TC-PTP), encoded by *Ptpn2*, in chemically-induced skin carcinogenesis *via* the negative regulation of STAT3 and AKT signaling. Using epidermal specific TC-PTP knockout (*K14Cre.Ptpn2*^*fl/fl*^) mice, we demonstrate loss of TC-PTP led to a desensitization to tumor initiator 7,12-dimethylbenz[a]anthracene (DMBA)-induced apoptosis both *in vivo* epidermis and *in vitro* keratinocytes. TC-PTP deficiency also resulted in a significant increase in epidermal thickness and hyperproliferation following exposure to the tumor promoter, 12-*O*-tetradecanoylphorbol-13-acetate (TPA). Western blot analysis showed that both phosphorylated STAT3 and phosphorylated AKT expressions were significantly increased in epidermis of TC-PTP-deficient mice compared to control mice following TPA treatment. Inhibition of STAT3 or AKT reversed the effects of TC-PTP deficiency on apoptosis and proliferation. Finally, TC-PTP knockout mice showed a shortened latency of tumorigenesis and significantly increased numbers of tumors during two-stage skin carcinogenesis. Our findings reveal that TC-PTP has potential as a novel target for the prevention of skin cancer through its role in the regulation of STAT3 and AKT signaling.

T-cell protein tyrosine phosphatase (TC-PTP; encoded by *PTPN2*), one of 17 intracellular and non-receptor PTPs, was originally cloned from a human T-cell cDNA library. TC-PTP is ubiquitously expressed in embryonic and adult tissues, although it is highly expressed in hematopoietic tissues^1^,^2^. Alternative splicing at the 3′ end of the *PTPN2* gene generates two distinct forms of TC-PTP: TC45 (TC-PTPa) and TC48 (TC-PTPb). TC45 (45 kDa) is primarily expressed in the nucleus with a bipartite nuclear localization signal in its C terminus, whereas TC48 (48 kDa) is localized to the endoplasmic reticulum with a hydrophobic C terminus^3^,^4^. TC-PTP is involved in the regulation of various physiological functions including cell cycle regulation and apoptosis through dephosphorylation of its target substrates, such as JAK1, JAK3, STAT1, STAT3 and STAT5^5^,^6^.

Generation of TC-PTP knockout mice showed its critical role in hematopoiesis and immune function in that TC-PTP knockout mice were severely defective in the hematopoietic compartment and all homozygous mice died between 3 and 5 weeks of age due to diarrhea, splenomegaly, lymphadenopathy, and anemia^7^. Recent studies also showed that TC-PTP has a critical role in the regulation of diabetes and obesity through its ability to modulate insulin and leptin signaling^8^. Neuronal cell-specific TC-PTP-deficient mice showed reduced high-fat diet-induced weight gain and enhanced leptin sensitivity with increased STAT3 phosphorylation in the hypothalamus after leptin administration, indicating that TC-PTP is involved in the development of leptin resistance^9^. Furthermore, proopiomelanocortin (POMC) neuron cell-specific TC-PTP-deficient mice showed enhanced AKT signaling and POMC expression in the arcuate nucleus of the hypothalamus in response to insulin, suggesting that TC-PTP attenuates insulin signaling in POMC neurons^10^.

In regards to cancer, recent studies have shown that focal deletion of *PTPN2* was detected in human T-cell acute lymphoblastic leukemia, implying TC-PTP has the potential to act as a tumor suppressor^11^. Studies also have shown that TC-PTP has a tumor suppressive function in breast and colorectal cancers mainly by regulating STAT3 signaling. The level of TC-PTP expression was decreased in a subset of breast cancer cell lines and a large proportion of triple-negative primary human breast cancers^12^,^13^. TC-PTP overexpression in human breast cancer cell lines suppressed cell proliferation and anchorage-independent growth with reduced tyrosine phosphorylation of STAT3 and SRC family kinase^12^. GdX (X-linked gene in the *G6PD* cluster at Xq28), which is known to act as a chaperon in protein processing in the endoplasmic reticulum, stabilizes the steady-state association of phosphorylated STAT3 with TC45 and promotes STAT3 dephosphorylation. Deletion of *GdX* in mice significantly accelerated colitis-associated colorectal tumorigenesis that corresponded with an increase in the level of phosphorylated STAT3^13^.

Initial studies of PTPs in skin showed that despite the fact that PTP expression is induced during keratinocyte proliferation and maturation, their expression levels remain unchanged within epidermal tissue^14^. Exposure to acute ultraviolet (UV) irradiation or treatment with the tumor promoter, 12-*O*-tetradecanoylphorbol-13-acetate (TPA), increases the activation of PTKs including the epidermal growth factor receptor (EGFR) and the downstream STAT3 signaling pathway^15^,^16^,^17^,^18^,^19^,^20^. One possible explanation for this result is that UV irradiation or TPA treatment induces PTP inactivation and in fact, studies revealed that reactive oxygen species produced by UV irradiation or TPA treatment triggered the inactivation of PTPs by oxidizing the cysteine residue within the conserved active-site of the PTP catalytic domain^21^,^22^,^23^. However, our recent studies showed that STAT3 is initially dephosphorylated in keratinocytes in response to UVB irradiation, and treatment with Na_3_VO_4_, a pan PTP inhibitor, recovered the level of phosphorylated STAT3^18^. Furthermore, our studies demonstrated that TC-PTP is one PTP that is responsible for UVB-mediated STAT3 dephosphorylation in skin^24^. UVB-mediated activation of TC-PTP resulted in a significant decrease in cell proliferation corresponding with a decrease of STAT3 phosphorylation in mouse keratinocytes^25^, suggesting that TC-PTP-mediated signaling may serve as part of a protective mechanism against skin carcinogenesis.

In the current study, we show a crucial role for TC-PTP in attenuating chemically-induced skin cancer formation. TPA initially increased STAT3 dephosphorylation and TC-PTP deficiency significantly reduced the effect of TPA on STAT3 dephosphorylation in mouse keratinocytes. *In vivo* studies using epidermal-specific TC-PTP knockout mice revealed that loss of TC-PTP significantly reduced 7,12-dimethylbenz[a]anthracene (DMBA)-induced apoptosis and increased TPA-induced cell proliferation mainly through the regulation of STAT3 and AKT phosphorylation, which resulted in enhanced skin cancer formation during DMBA/TPA skin carcinogenesis. These results suggest that TC-PTP plays a protective role against chemically-induced skin carcinogenesis.

## Results

### Knockdown of TC-PTP leads to reduced STAT3 dephosphorylation in mouse keratinocytes following TPA treatment

**Figure 1:**
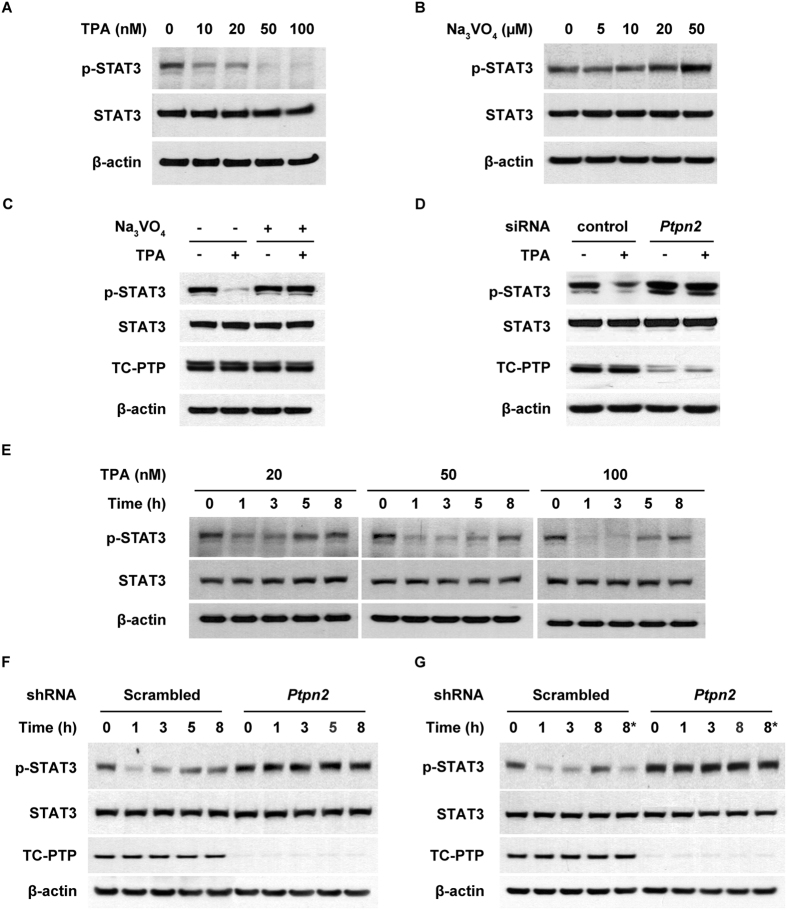
Effects of TC-PTP deficiency on STAT3 dephosphorylation in keratinocytes following TPA treatment. Total cell lysates were resolved by SDS-PAGE and immunoblotted with antibodies specific for STAT3 and phosphorylated STAT3 (p-STAT3). (**A**) 3PC keratinocytes were treated with 10, 20, 50 and 100 nM of TPA for 1 h. (**B**) 3PC keratinocytes were cultured in the presence of 5, 10, 20 and 50 μM Na_3_VO_4_ for 6 h. (**C**) 3PC keratinocyte were cultured with 50 μM of Na_3_VO_4_ for 2 h before treatment with 100 nM TPA. Cells were collected 6 h after TPA treatment. (**D**) 3PC keratinocytes were transfected with siRNA specific for *Ptpn2.* Cells were collected 1 h after TPA (100 nM) treatment. (**E**) 3PC keratinocytes were treated with 20, 50 and 100 nM of TPA for 1 h. Cells were collected at the indicated time after TPA treatment. (**F**–**G**) Control (scrambled) and TC-PTP-knockdown keratinocytes were treated with TPA (20 nM) for 1 h. Following TPA treatment, cells were collected at the indicated time. (**G**) *At 3 h of incubation, control and TC-PTP-knockdown keratinocytes were re-treated with TPA for 1 h. Cells were then collected 4 h after second TPA treatment.

We previously showed that PTPs are involved in rapid STAT3 dephosphorylation in keratinocytes in response to UVB exposure^24^. Of the three PTPs involved in UVB-mediated STAT3 dephosphorylation, TC-PTP has a major role in regulating STAT3 signaling and significantly suppresses keratinocyte survival and proliferation following UVB irradiation^25^. In the current studies, we examined whether treatment with TPA, a potent tumor promoter, can result in rapid dephosphorylation of STAT3 because STAT3 plays a critical role in chemically-induced skin carcinogenesis^26^,^27^,^28^,^29^. Furthermore, we examined whether TC-PTP is involved in this mechanism given that TC-PTP can regulate STAT3 signaling. As shown in Fig. 1A, the level of phosphorylated STAT3 in mouse keratinocytes was decreased in response to TPA treatment in a dose-dependent manner. To demonstrate PTP is involved in this response, keratinocytes were cultured with Na_3_VO_4_. The level of phosphorylated STAT3 increased with higher doses of Na_3_VO_4_ (Fig. 1B). In addition, pretreatment with Na_3_VO_4_ for 2 h before TPA exposure recovered the level of STAT3 phosphorylation that was inhibited by TPA (Fig. 1C). To examine whether TC-PTP specifically is involved in TPA-induced STAT3 dephosphorylation, TC-PTP was knocked down using siRNA before TPA treatment. As shown in Fig. 1D, the level of STAT3 phosphorylation after TPA treatment in control keratinocytes transiently transfected with scrambled siRNA was significantly reduced compared to untreated control. However, the level of STAT3 phosphorylation after TPA treatment in keratinocytes transiently transfected with *Ptpn2*-specific siRNA was not reduced compared to untreated *Ptpn2*-deficient keratinocytes, indicating that TC-PTP is involved in the regulation of STAT3 phosphorylation following TPA exposure. To further examine the effect of TPA on STAT3 dephosphorylation in keratinocytes, cells were treated with TPA for 1 h and then harvested at the indicated time (Fig. 1E). Following the one hour pulse treatment of TPA, the level of phosphorylated STAT3 rapidly decreased but then recovered at later time points (Fig. 1E), which is similar to the results previously observed in keratinocytes after exposure to a low dose of UVB^25^. However, the level of phosphorylated STAT3 in a stable TC-PTP-knockdown keratinocyte cell line was not reduced by pulse treatment of TPA (Fig. 1F). During the promotion stage of skin carcinogenesis, initiated cells undergo clonal expansion induced by repeated exposure to a tumor promoter, which eventually leads to the development of benign tumors. Therefore, we treated keratinocytes with TPA for 1 h and then treated cells again 3 h after initial exposure to test if TC-PTP can be reactivated by repeated TPA treatments. A single treatment of TPA initially reduced the expression of phosphorylated STAT3 in control keratinocytes but then expression recovered after 8 h. However, re-treatment of the control cells with TPA after 3 h decreased phosphorylated STAT3 again (Fig. 1G). Furthermore, the second treatment of TPA effectively decreased the level of phosphorylated STAT3 beyond that of a single TPA treatment. The same effect was not seen in TC-PTP-knockdown keratinocytes (Fig. 1G). Together, these results suggest that TC-PTP plays a major role in the regulation of STAT3 in the cellular response to TPA exposure and this response can serve as one critical protective mechanism against aberrant STAT3 activation by an environmental toxin.

### Generation of skin specific *Ptpn2* conditional knockout mice

**Figure 2:**
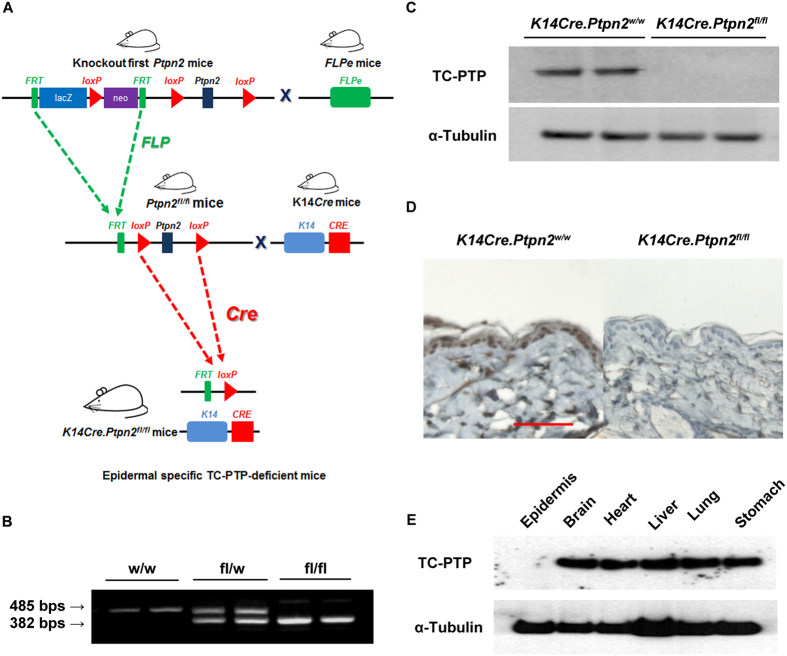
Generation of epidermal-specific TC-PTP knockout mice. (**A**) Schematic diagram of targeted disruption of the *Ptpn2* gene within the epidermis using the *Cre-LoxP* system. (**B**) PCR analysis of the *Ptpn2* deletion using genomic DNA obtained from the mouse tail. Allele-specific PCR was performed using the three primers described in Materials and Methods to evaluate the efficiency of the *Ptpn2* deletion. (**C**) Western blot analysis of TC-PTP in the epidermis from *K14Cre.Ptpn2*^*w/w*^ and *K14Cre.Ptpn2*^*fl/fl*^ mice. Epidermis was collected and total cell lysates from both genotypes were resolved by SDS-PAGE and immunoblotted with antibodies specific for TC-PTP. (**D**) Immunohistochemical staining of TC-PTP in the epidermis from both genotypes. (**E**) Western blot analysis of TC-PTP in non-epidermal tissues from *K14Cre.Ptpn2^fl/fl^* mice. Brain, heart, liver, lung, and stomach were collected from *K14Cre.Ptpn2^fl/fl^* mice and total cell lysates were prepared.

Early lethality of TC-PTP knockout mice (~3–5 weeks of age) makes it impossible to characterize the functional role of TC-PTP in carcinogenesis using this mouse model^7^. Therefore, for this study, we generated epidermal specific TC-PTP knockout mice to further elucidate the protective role TC-PTP may play in chemically-induced skin cancer formation. To generate our mouse model, we obtained ‘Knockout (KO)-first allele’ transgenic mice established on a C57BL/6 congenic background that carry a floxed *Ptpn2* allele from the EUCOMM/IMPC. We bred these ‘KO-first allele’ mice with FLPe mice (obtained from The Jackson Laboratory) to delete the targeting cassette including the splice acceptor gene and reporter gene and derive “conditional” *Ptpn2* mice. We also obtained *K14Cre* transgenic mice established on a C57BL/6 congenic background that express human keratin 14-conrolled Cre recombinase in the basal cell layer of the epidermis and hair follicles from The Jackson Laboratory. The C57BL/6 strain is not suitable for carcinogenesis studies because of its strong resistance to chemical carcinogens. The FVB/N strain, on the other hand, is highly sensitive to chemically-induced skin carcinogenesis in comparison to other strains, such as C57BL/N^30^. Therefore, we backcrossed both “conditional” *Ptpn2* mice and *K14Cre* mice against wild-type mice of FVB/N background for at least 10 generations. Then, conditional *Ptpn2* mice were bred with *K14Cre* mice to generate epidermal specific TC-PTP knockout (KO, *K14Cre.Ptpn2*^*fl/fl*^) mice (Fig. 2A). These TC-PTP KO mice were identified by genotyping with primers specific for *Ptpn2* wild-type allele and *Ptpn2* floxed allele which produced a 485 bp PCR product or a 382 bp PCR product, respectively (Fig. 2B). Western blot and immuohistochemical analysis clearly showed that TC-PTP KO mice do not express TC-PTP in the epidermis in contrast to control (WT, *K14Cre.Ptpn2*^*w/w*^) mice (Fig. 2C,D). We also isolated protein from other tissues of TC-PTP KO mice and found TC-PTP was expressed normally in all other tissues except epidermis, demonstrating epidermal specific deletion of *Ptpn2* by Cre recombinase (Fig. 2E).

### TC-PTP deficiency increases resistance to apoptosis

**Figure 3:**
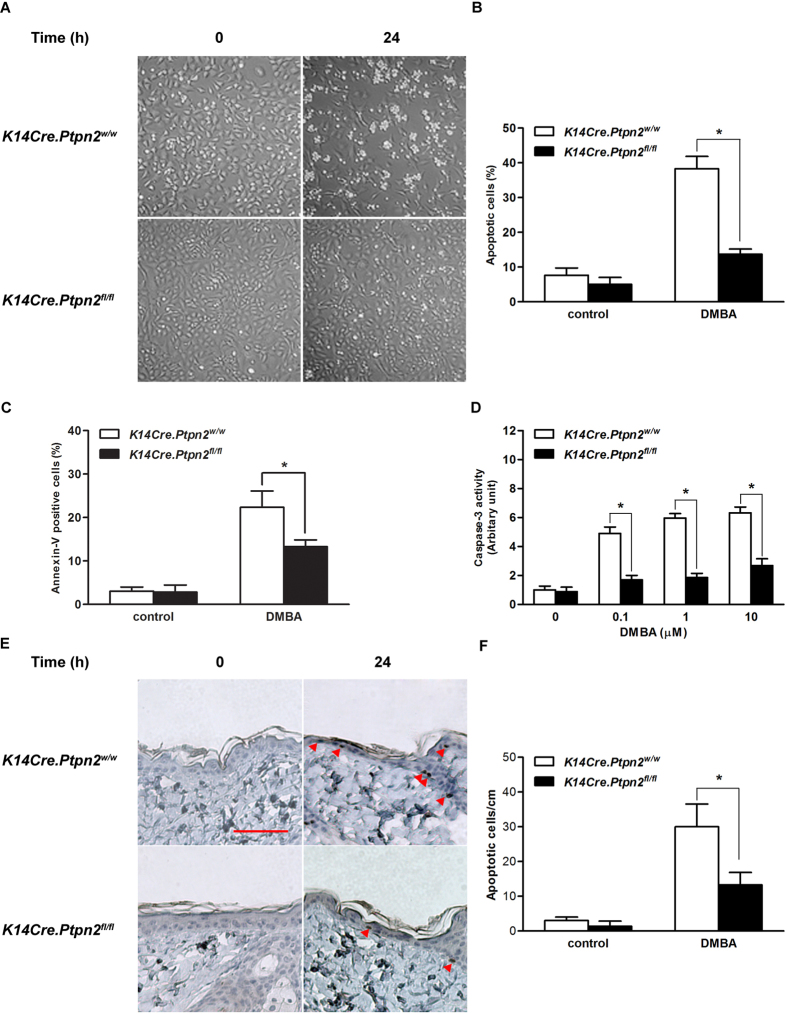
Effect of TC-PTP deletion on DMBA-induced apoptosis. (**A**–**C**) Apoptotic response of primary keratinocytes obtained from the epidermis of *K14Cre.Ptpn2*^*w/w*^ and *K14Cre.Ptpn2*^*fl/fl*^ mice after 24 h of DMBA treatment. (**A**) Primary keratinocytes from both genotypes were cultured and treated with 100 nM of DMBA. (**B**) Quantitative analysis of percentage of apoptotic cells characterized by cell ballooning, nuclear condensation, and bleb formation. After 24 h of DMBA treatment, apoptotic keratinocytes were counted microscopically in at least three non-overlapping fields. Results are the mean ± s.d.m. from three independent experiments. *p < 0.05 by Mann-Whitney *U* test. (**C**) Flow cytometry analysis for apoptosis with Annexin V-FITC was measured after 24 h of 100 nM DMBA treatment. *p < 0.05 by Mann-Whitney *U* test. (**D**) Caspase-3 activity was measured after 24 h of DMBA treatment. *p < 0.05 by Mann-Whitney *U* test. (**E**–**F**) Apoptotic response of the epidermis from both genotypes. Groups of mice (n = 3) received a single topical application of DMBA (200 nmol) and sacrificed 24 h later. Skin sections were collected and apoptotic cells were quantified by immunostaining with caspase-3. (**E**) Representative staining of caspase-3 in the epidermis from both genotypes following treatment with DMBA. Scale bar: 100 μm. (**F**) Quantitative analysis of caspase-3-positive cells per centimeter of epidermis in both genotypes after DMBA treatment. *p < 0.05 by Mann-Whitney *U* test.

In chemically-induced skin carcinogenesis, DNA damage caused by a carcinogen, such as DMBA, can be repaired by cellular DNA repair mechanisms otherwise, apoptotic signaling may be activated as protection against propagation of aberrant, damaged cells. However, damaged cells can acquire a growth advantage through carcinogen-induced mutation(s) during tumor initiation, if they have escaped these protective mechanisms, and these cells can clonally expand as a result of selective pressure by repetitive exposure to a tumor promoter, such as TPA^31^,^32^. To investigate the effect of TC-PTP deficiency on DMBA-induced apoptosis, primary keratinocytes from both *K14Cre.Ptpn2*^*w/w*^ and *K14Cre.Ptpn2*^*fl/fl*^ mice were cultured and treated with DMBA. As shown in Fig. 3A, there are no visible differences in morphology between TC-PTP WT and TC-PTP KO keratinocytes before DMBA treatment. However, 24 h after DMBA treatment, profound morphological changes induced by apoptosis, such as cell ballooning and bleb formation, were observed in control keratinocytes and loss of TC-PTP significantly reduced the apoptotic effect (Fig. 3A,B). Further analysis using flow cytometry showed that annexin V-positive cells were significantly increased wild-type keratinocytes compared to TC-PTP-deficient keratinocytes (Fig. 3C). Caspase-3 activity was also increased in wild-type primary keratinocytes following DMBA treatment whereas DMBA had little to no effect on caspase-3 activity in TC-PTP-deficient keratinocytes (Fig. 3D) as evidenced by the significant difference in activity between control and TC-PTP-deficient cells following treatment. Consistent with our *in vitro* results, the number of apoptotic cells, detected by active caspase-3 staining, within the epidermis of control mice was significantly increased compared to TC-PTP-deficient mouse epidermis 24 h after DMBA treatment (Fig. 3E,F). These results suggest that TC-PTP has a protective function in epidermis in that it mediates the removal of DNA-damaged keratinocytes following DMBA treatment.

### Role of TC-PTP in TPA-induced epidermal hyperproliferation and hyperplasia

**Figure 4:**
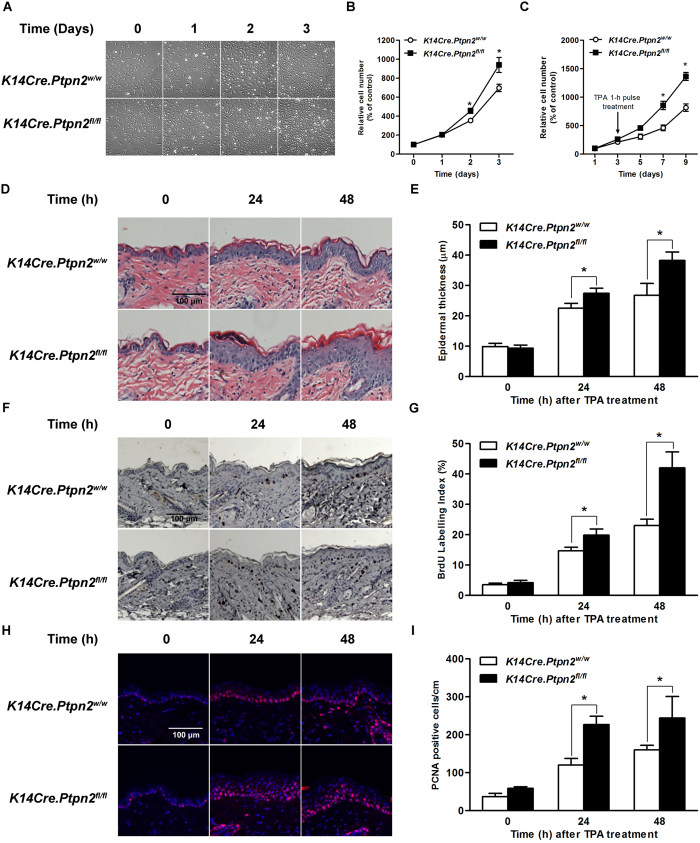
Effect of TC-PTP deficiency on TPA-induced epidermal proliferation. (**A**) Representative photomicrographs of primary keratinocytes from *K14Cre.Ptpn2*^*w/w*^ and *K14Cre.Ptpn2*^*fl/fl*^ mice after days 1–3 of culture. Equivalent number of primary keratinocytes from both genotypes were seeded and cultured for 3 days. (**B**) Proliferation of primary keratinocytes was measured using the WST-assay. *p < 0.05 by Mann-Whitney *U* test. (**C**) Primary keratinocytes were treated with TPA (40 nM) for 1 h. Proliferation of primary keratinocytes was measured using WST-assay. (**D–I**) Groups of 7 weeks old mice (n = 3/group) were treated topically with single application of TPA (6.8 nmol) and sacrificed 24 or 48 h after treatment. (**D**) Representative hematoxylin and eosin staining of epidermis from both genotypes of mice. Scale bar: 100 μm. (**E**) Quantification of epidermal thickness from both genotypes treated with TPA. *p < 0.05 by Mann-Whitney *U* test. (**F**) Representative BrdU staining of epidermis from both genotypes of mice. Scale bar: 100 μm. (**G**) Quantification of BrdU-labled keratinocytes from epidermis of both genotypes. *p < 0.05 by Mann-Whitney *U* test. (**H**) Representative PCNA staining of epidermis from both genotypes of mice. Scale bar: 100 μm. (**I**) Quantification of PCNA-positive keratinocytes from epidermis of both genotypes. *p < 0.05 by Mann-Whitney *U* test.

As previously mentioned, cells which survive the initiation stage confer a growth advantage and stimulate keratinocyte proliferation, resulting in epidermal hyperplasia during the tumor promotion stage. To investigate the effect of TC-PTP deficiency on TPA-induced keratinocyte proliferation and epidermal hyperplasia, primary keratinocytes derived from *K14Cre.Ptpn2*^*w/w*^ and *K14Cre.Ptpn2*^*fl/fl*^ mice were cultured. Interestingly, TC-PTP-deficient keratinocytes significantly grew faster (~1.5-fold, *P* < 0.05) than control keratinocytes (Fig. 4A,B). Following 1 h pulse treatment of TPA, keratinocytes from both genotypes exhibited an initial decrease in growth rate (Fig. 4C). After the short lag period, both keratinocytes began to grow again, but TC-PTP-deficient keratinocytes proliferated significantly faster (~3-fold, *P* < 0.05) than control keratinocytes.

To further investigate whether loss of TC-PTP confers a proliferative advantage in response to TPA on epidermal cells *in vivo*, both TC-PTP WT and TC-PTP KO mice were treated with TPA. As shown in Fig. 4D,E, there were no significant differences in the epidermal thickness of the two genotypes. However, application of TPA to the epidermis remarkably increased epidermal thickness in TC-PTP KO mice compared to control mice 24 and 48 h after treatment. BrdU labeling and PCNA staining, which measure active cell proliferation, showed a similar effect for TPA on epidermal thickness. Following TPA treatment, the number of either BrdU-positive or PCNA-positive cells in the epidermis of TC-PTP KO mice was significantly increased compared to control mice (Fig. 4F–I). These results reveal that TC-PTP can play a role in protecting mouse epidermis against TPA-induced epidermal hyperproliferation.

### Loss of TC-PTP confers higher proliferation and resistance against DMBA-induced apoptosis through STAT3 and AKT pathways

**Figure 5:**
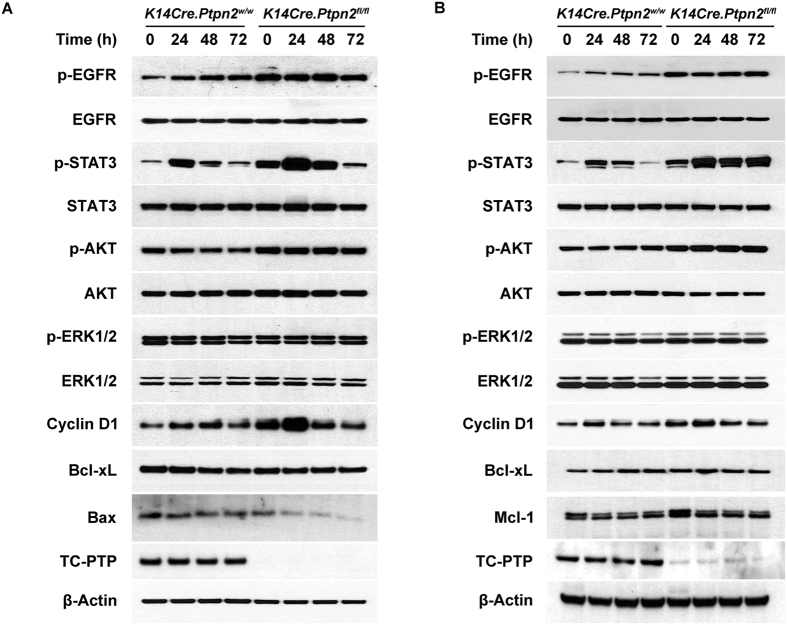
TC-PTP deficiency confers a resistance against DMBA-induced apoptosis and an enhanced TPA-induced cell proliferation through the regulation of STAT3 and AKT signaling. (**A**) Western blot analysis of p-EGFR, p-STAT3, p-AKT, p-ERK1/2, cyclin D1, Bcl-xL, Bax in the epidermis from *K14Cre.Ptpn2*^*w/w*^ and *K14Cre.Ptpn2*^*fl/fl*^ mice after TPA treatment. Mice were sacrificed at the indicated time after TPA treatment and epidermal cell lysates were prepared. (**B**) Western blot analysis of p-EGFR, p-STAT3, p-AKT, p-ERK1/2, cyclin D1, Bcl-xL, Mcl-1 in primary keratinocytes from both genotypes after TPA treatment. Cells were collected at the indicated time after TPA treatment and total cell lysates were prepared.

**Figure 6:**
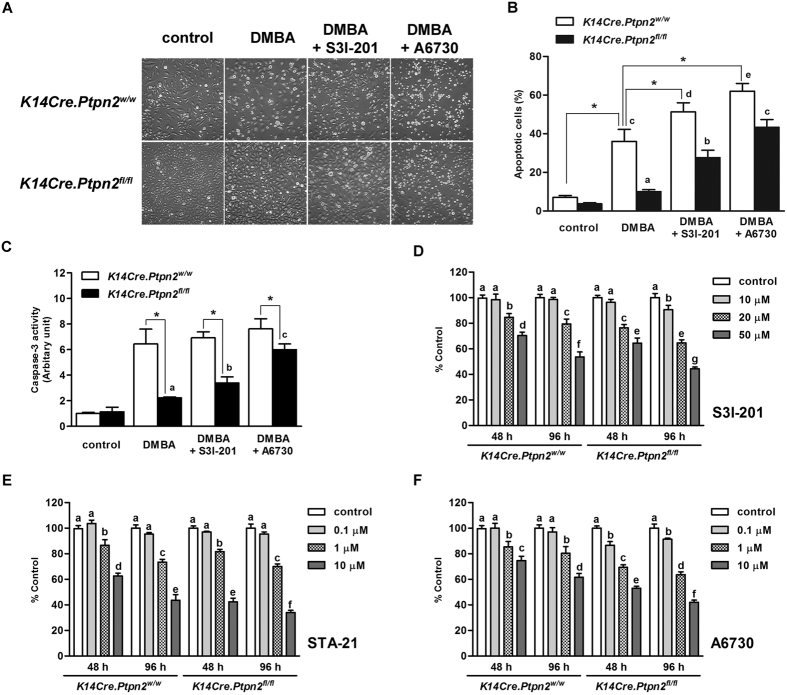
Inhibition of STAT3 or AKT on keratinocyte survival and proliferation. (**A**–**C**) Effect of inhibition of STAT3 or AKT on DMBA-induced apoptosis in keratinocytes. Primary keratinocytes from both genotypes were pretreated with either STAT3 inhibitor S3I-201 (20 μM) or AKT1/2 inhibitor A6730 (10 μM) for 1 h before DMBA treatment. (**A**) Representative images of morphological changes of primary keratinocytes from both genotypes following treatment of DMBA for 24 h. (**B**) Quantitative analysis of percentage of apoptotic cells after 24 h of DMBA treatment. *p < 0.05 by Mann-Whitney *U* test. Values labelled (**A**-**E**) were evaluated against each other by ANOVA; bars designated by the same letter are statistically similar, whereas bars designated with *different letters* are significantly different, *p* < 0.05. (**C**) Caspase-3 activity was measured after 24 h of DMBA treatment. *p < 0.05 by Mann-Whitney *U* test. Values designated by the same letter are statistically similar, whereas values designated with *different letters* are significantly different at *p* < 0.05 as determined by ANOVA. (**D**–**F**) Effect of inhibition of STAT3 or AKT on TPA-induced cell proliferation in keratinocytes. Primary keratinocytes from both genotypes were pretreated with either AKT1/2 inhibitor A6730 (10 μM) or STAT3 inhibitor S3I-201 (20 μM) for 1 h before TPA treatment. Following the 1 h pulse treatment of TPA (40 nM), cell proliferation was measured at the indicated time using WST-assay. (**D**) Inhibition of cell proliferation by S3I-201 (10–50 μM). (**E**) Inhibition of cell proliferation by STA-21 (0.1–10 μM). (**F**) Inhibition of cell proliferation by A6730 (0.1–10 μM). Values designated by the same letter are statistically similar, whereas values designated with *different letters* are significantly different, *p* < 0.05 determined by ANOVA.

We have shown previously and in our current study that TC-PTP dephosphorylates STAT3 (Fig. 1). EGFR is another substrate of TC-PTP that has a major role in cell growth^33^, consequently, TC-PTP negatively regulates PI3K/AKT and MAPK signaling pathways, which are downstream signaling pathways of EGFR^10^,^34^,^35^,^36^,^37^. Furthermore, these four signaling pathways – STAT3, EGFR, PI3K/AKT, and MAPK/ERK – are major pathways that are involved in skin carcinogenesis^38^. To investigate TC-PTP-mediated regulation of these signaling pathways during tumor promotion, both *K14Cre.Ptpn2*^*w/w*^ and *K14Cre.Ptpn2*^*fl/fl*^ mice were treated with TPA and epidermal cell lysates were prepared for western blot analysis. As shown in Fig. 5A, the level of phosphorylated STAT3 was higher in the epidermis of TC-PTP KO mice compared to that of control mice. In response to TPA treatment, the level of phosphorylated STAT3 was increased in both control and TC-PTP KO mice but the levels were higher in TC-PTP KO mice than control mice, especially 24 and 48 h after TPA treatment. The level of cyclin D1 expression, one critical target of STAT3, also was increased in both genotypes and its expression levels were higher in TC-PTP KO mice than control mice. However, the level of Bcl-xL expression was not changed in either genotypes following TPA treatment, indicating that Bcl-xL expression is independent of STAT3 and TC-PTP. Similar effects on Bcl-xL expression were observed in UVB-irradiated keratinocytes^24^. In addition, the level of phosphorylated EGFR was higher in TC-PTP KO mice than control mice in the absence or presence of TPA treatment. Similarly, the level of phosphorylated AKT was higher in TC-PTP KO mice compared to control mice with or without TPA treatment. The level of Bax expression, an apoptotic downstream target of AKT^39^, was also lower in TC-PTP KO mice compared to control mice. Interestingly, phosphorylated ERK1/2 was similarly expressed in both types of mice. We confirmed our *in vivo* results using primary keratinocytes derived from both genotypes (Fig. 5B). The levels of phosphorylated STAT3 and phosphorylated AKT were higher in TC-PTP-deficient keratinocytes compared to control keratinocytes with or without TPA treatment. The level of Mcl-1 expression, an anti-apoptotic downstream target of AKT^40^,^41^, was also higher in TC-PTP-deficient keratinocytes compared to control keratinocytes. TC-PTP deficiency has no effect on ERK1/2 phosphorylation in keratinocytes, as we observed with epidermis. To further examine whether TC-PTP-mediated regulation of STAT3 and AKT signaling has effects on DMBA-induced apoptosis, both wild-type and TC-PTP-deficient primary keratinocytes were treated with either a STAT3-specific inhibitor (S3I-201) or an AKT-specific inhibitor (A6730) before DMBA treatment. As shown in Fig. 6A and B, inhibition of STAT3 or AKT significantly increased cell sensitivity to DMBA-induced apoptosis in both control and TC-PTP-deficient keratinocytes, and the increased extent was higher in TC-PTP-deficient keratinocytes compared to control keratinocytes. In addition, DMBA-induced caspase-3 activity was significantly increased by inhibition of STAT3 or AKT in TC-PTP-deficient keratinocytes in comparison to DMBA-only control (Fig. 6C; a,b,c). Interestingly, inhibition of STAT3 or AKT in TC-PTP-deficient keratinocytes could not completely recover the apoptotic effect of DMBA to the levels seen in wild-type keratinocytes (Fig. 6A–C), suggesting STAT3 and AKT have a synergistic effect and/or TC-PTP has an additional role(s) in DMBA-induced apoptosis. Additionally, we investigated the effects of STAT3 and AKT inhibition on TPA-induced keratinocyte proliferation. Both control and TC-PTP-deficient keratinocytes were treated with either STAT3 or AKT inhibitor before TPA treatment and then the number of cells were counted after seven days of TPA treatment. Inhibition of either STAT3 or AKT significantly decreased TPA-induced cell proliferation in TC-PTP-deficient keratinocytes compared to control keratinocytes (Fig. 6D–F). Our results suggest that TC-PTP helps protect keratinocytes against tumor initiation and progression by facilitating DMBA-induced apoptosis and inhibiting TPA-induced cell proliferation *via* the negative regulation of STAT3 and AKT signaling.

### Disruption of TC-PTP in epidermis significantly enhances skin tumor development

**Figure 7:**
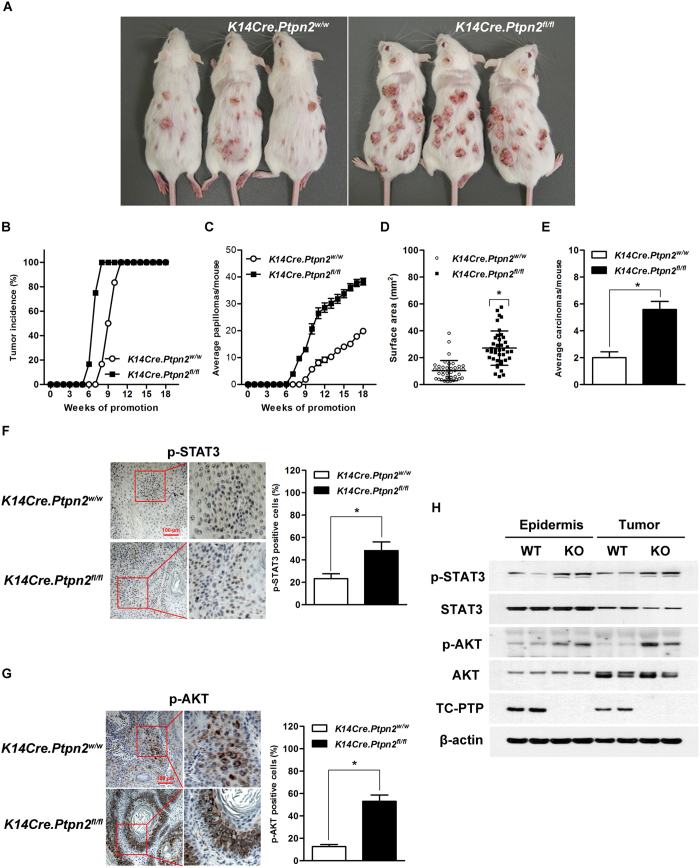
Effect of TC-PTP disruption in the epidermis during two-stage skin carcinogenesis. Groups of *K14Cre.Ptpn2*^*fl/fl*^ and *K14Cre.Ptpn2*^*w/w*^ mice (n = 12) were treated with 25 nmol DMBA and after 2 weeks treated with twice-weekly application of 6.8 nmol of TPA for the duration of the experiment. (**A**) Representative photograph of *K14Cre.Ptpn2*^*w/w*^ and *K14Cre.Ptpn2*^*fl/fl*^ mice at the 18^th^ week of tumor promotion. (**B**) Percentage of mice with tumors. (**C**) Average number of tumors per mouse. (mean ± s.e.m.). (**D**) Tumor size. The surface area of tumors was measured at the 18^th^ week of tumor promotion. *p < 0.05 by Mann-Whitney *U* test. (**E**) Average carcinomas per mouse. (mean ± s.e.m.). *p < 0.05 by Mann-Whitney *U* test. (**F**–**G**) Representative immunohistochemical staining of p-STAT3, and p-AKT in papillomas from both genotypes. The values are represented as a mean ± s.d.m. (n = 5). *p < 0.05 by Mann-Whitney *U* test. Scale bar: 100 μm. (**F**) p-STAT3 staining. Right, quantitative analysis of percentage of p-STAT3-positive cells in papillomas. (**G**) p-AKT staining. Right, quantitative analysis of percentage of p-STAT3-positive cells in papillomas. (**H**) Expression of p-STAT3 and p-AKT in the epidermis and papillomas from both genotypes. Epidermal cell lysates were isolated from both genotypes (untreated). Five to 10 papillomas were collected and pooled at the end of the study to generate protein lysates.

To further demonstrate the protective role of TC-PTP during chemically-induced skin carcinogenesis, TC-PTP KO and control mice were subjected to a two-stage chemical carcinogenesis regimen. Both groups of mice were treated with DMBA. Two weeks after DMBA initiation, mice were treated with TPA twice weekly. TC-PTP KO mice were more sensitive to chemically-induced skin carcinogenesis in comparison to control mice (Fig. 7A–D). Even though 100% of both groups of mice developed papillomas by the end of experiments, TC-PTP KO mice developed papillomas faster than control mice. TC-PTP KO mice started to develop papilllomas 6 weeks after promotion, whereas control mice did not develop papillomas until 8 weeks after promotion. Even after 8 weeks, only 17% of the control group had developed papillomas while 100% of the TC-PTP KO group had already developed papillomas (Fig. 7B). Furthermore, the average number of papillomas/tumors per mouse was significantly greater (more than 2-fold) in TC-PTP KO mice compared with control mice (Fig. 7C). The average size of papillomas/tumors and the average number of carcinomas per mouse were also significantly greater in TC-PTP-deficient mice compared with control mice at the end of the experiments (Fig. 7D,E). Immunohistochemical and western blot analyses clearly showed that papillomas from control mice expressed TC-PTP, whereas papillomas from TC-PTP KO mice did not (Fig. 7H). In addition, papillomas from TC-PTP KO mice had significantly higher levels of phosphorylated STAT3 and phosphorylated AKT expression when compared with control (Fig. 7F–H). Collectively, our findings indicate that TC-PTP mediates a protective mechanism that is able to attenuate chemically-induced skin carcinogenesis and its function is associated with the STAT3 and AKT signaling pathways.

## Discussion

Phosphotyrosine-based signaling is regulated by the balanced actions of PTKs and PTPs, and it is involved in the regulation of various cellular processes including growth, differentiation, metabolism, and motility^42^,^43^. The aberrant activation of signaling pathways by mutation and/or overexpression of PTKs can lead to various types of cancers including skin cancer^44^,^45^. In this regard, PTKs have been considered to be attractive targets for cancer prevention and have been extensively studied in skin carcinogenesis. However, PTPs, the homeostatic counterpart of PTKs, have not been studied in skin carcinogenesis since environmental toxins, such as UV radiation or chemical toxicants, can cause PTP inactivation by attacking the conserved active site within the PTP catalytic domain through reactive oxygen species^22^,^23^,^46^. In the current study, we demonstrate for the first time that TC-PTP can play a role in the prevention of skin cancer formation induced by DMBA/TPA carcinogenesis regimen through the use of our epidermal specific TC-PTP knockout mouse model. Knockdown of TC-PTP in mouse keratinocytes significantly recovered the level of activated STAT3 that was reduced by treatment with TPA. We show that loss of TC-PTP promotes resistance to DMBA-induced apoptosis and TPA-induced hyperproliferation within both *in vitro* primary keratinocytes and *in vivo* epidermis through the regulation of STAT3 and AKT signaling pathways. Furthermore, TC-PTP deficiency in epidermis significantly increased the number of papillomas/tumors and accelerated onset of tumor formation during two-stage skin carcinogenesis.

Previous studies showed that three PTPs – TC-PTP, SHP1, and SHP2 – cooperate to dephosphorylate STAT3 following UVB irradiation of mouse keratinocytes^24^. Further studies revealed that, of the three PTPs, TC-PTP is the major regulator of STAT3 signaling in keratinocytes during the response to UVB^25^. However, SHP1 and SHP2 may still contribute to STAT3 regulation or may be able to compensate for loss of TC-PTP. For instance, the previous studies demonstrated that following UVB exposure, levels of STAT3 phosphorylation in keratinocytes were quickly reduced but able to recover over time, with TC-PTP-knockdown cells expressing a higher level of active STAT3 compared to control; yet, even after recovery, the levels of phosphorylated STAT3 in TC-PTP-knockdown cells with and without UVB treatment were not the same. Also, the level of phosphorylated STAT3 after treatment with a low dose of UVB more rapidly recovered in TC-PTP-knockdown cells compared with control cells. In this study, we found that while the level of phosphorylated STAT3 was significantly decreased in control cells following TPA treatment, it was not decreased in TC-PTP-knockdown cells treated with TPA (Fig. 1D). Furthermore, TPA treatment did not induce a reduction of phosphorylated STAT3 in TC-PTP-knockdown cells, whereas it was initially decreased in similarly-treated control cells (Fig. 1F). These results suggest that the TPA-mediated mechanism of PTP activation is different from UVB. It is possible that either TPA activates only TC-PTP or it has a minimal effect on SHP1/SHP2 activation.

Our studies suggest that a single exposure to TPA does not constitutively activate TC-PTP. TC-PTP activation is reversible, as evidenced by the initial decrease and then recovery of phosphorylated STAT3 in keratinocytes after 1 h pulse treatment of TPA (Fig. 1F,G). However, re-treatment with TPA reduced phosphorylated STAT3 again, and this re-dephosphorylation of STAT3 was not observed in TC-PTP-knockdown cells, implying TC-PTP can be activated repeatedly by multiple applications of TPA (Fig. 1G). Our findings suggest that the reoccurring activation of TC-PTP during tumor promotion could continuously inhibit STAT3 phosphorylation/activation and thereby contribute to the prevention of skin cancer. It is the continuous activation of STAT3 by the repeated/long-term exposure to TPA that contributes to the development of skin cancer. We found that a single treatment of TPA induced expression of phosphorylated STAT3, 24 and 48 hours after exposure in both TC-PTP WT and TC-PTP KO epidermis and primary keratinocytes and then expression of phosphorylated STAT3 decreased to basal levels. However, loss of TC-PTP resulted in a greater increase in phosphorylated STAT3 (Fig. 5A,B). Moreover, TC-PTP deficiency in primary keratinocytes prevented the decrease in phosphorylated STAT3 following TPA treatment (72 h, Fig. 5B). The results imply that TC-PTP is important in regulating STAT3 activation in response to TPA exposure and without TC-PTP, TPA would have a greater and more rapid effect on mouse epidermis. We show that TC-PTP-deficient mice develop papillomas more quickly than wild-type control mice and this result corresponds with an increase in phosphorylated STAT3 expression. Still, redundant and/or compensatory mechanisms to TC-PTP regulatory signaling may exist in epidermis to provide additional protection against environmental exposure as evidenced by the decrease in phosphorylated STAT3 72 h after TPA treatment in TC-PTP KO cells (Fig. 5A).

In addition to STAT3, AKT is another critical downstream target of PTK signaling in skin carcinogenesis^47^,^48^,^49^,^50^. Mouse models expressing either epidermal specific AKT or constitutively active AKT showed a significantly increased sensitivity to two-stage skin carcinogenesis^50^. Studies also have shown that TC-PTP negatively regulates AKT signaling. Gene copy loss of *PTPN2* and lower mRNA levels in breast cancer was associated with AKT activation and poor prognosis^51^. Our studies showed that the levels of phosphorylated EGFR and phosphorylated AKT were constitutively higher in TC-PTP KO mice compared with control mice in the presence or absence of TPA treatment (Fig. 5A,B). Inhibition of AKT in TC-PTP-deficient keratinocytes significantly increased the sensitivity to DMBA-induced apoptosis and decreased cell proliferation induced by TPA (Fig. 6A–C,F). Also, the level of phosphorylated AKT was higher in papillomas developed from TC-PTP KO mice during two-stage skin carcinogenesis (Fig. 7G,H).

In conclusion, use of our epidermal specific TC-PTP knockout mouse model provided evidence that TC-PTP can play a role in attenuating chemically-induced skin cancer formation through its continuous or prolonged activation. TC-PTP activation can lead to a significant increase in apoptosis induced by the carcinogen DMBA, and it facilitates the inhibition of cell proliferation mediated by tumor promoter TPA. TC-PTP performs these functions, mainly, by negatively regulating STAT3 and AKT signaling pathways. Our findings imply that modulation of TC-PTP regulatory signaling, which is not yet defined, and/or use of TC-PTP activators could contribute to the prevention of skin carcinogenesis. Overall, these studies suggest that TC-PTP activation may be a novel therapeutic strategy for the prevention and treatment of skin cancer.

## Methods

### Generation of epidermal-specific TC-PTP knockout (*K14Cre.Ptpn2* ^*fl/fl*^) mice

Knockout-first *Ptpn2* mice (C57BL/6N; *Ptpn2*^*tm1a(EUCOMM)Wtsi*^) generated by the European Conditional Mouse Mutagenesis Program (EUCOMM)/the International Mouse Phenotyping Consortium (IMPC) were bred with FLPe mice (C57BL/6) (The Jackson Laboratory) to delete a splice acceptor/reporter cassette. The resultant conditional mice were identified by polymerase chain reaction of genomic DNA isolated from tail snips with two sets of primers: CAS, Ptpn2_F, Ptpn2_R and tm1c_F, tm1c_R, primers which confirmed the successful deletion of the cassette. Heterozygous conditional *Ptpn2*^*fl/w*^ mice were backcrossed for at least 10 generation onto FVB/N genetic background. *K14Cre* mice (The Jackson Laboratory), originally generated on C57BL/6 genetic background, were also backcrossed for at least 10 generation onto FVB/N genetic background. Then, *K14Cre* mice were crossed with *Ptpn2*^*fl/w*^ mice to generate a strain hemizygous for the *K14Cre* transgene and heterozygous for *Ptpn2* floxed allele. The resultant progeny were crossed to select for *K14Cre.Ptpn2*^*fl/fl*^ knockout mice and *K14Cre.Ptpn2*^*w/w*^ control mice. *Ptpn2* floxed gene was identified by polymerase chain reaction of genomic DNA isolated from tail snips with three primers: *CAS*_R1_Term (TCGTGGTATCGTTATGCGCC), *Ptpn2*_47877_F (GCCAAGAGACAGTGGAAAGAGAG), *Ptpn2*_47877_R (ACTGCAAAACCATAACTGGC). Primers *Ptpn2*_47877_F and *Cas*_R1_term generate a 382 bp fragment corresponding to floxed *Ptpn2* allele. Primers *Ptpn2*_47877_F and *Ptpn2*_47877_R generate a 485 bp fragment corresponding to wild-type *Ptpn2* allele. *Cre* gene was also identified by polymerase chain reaction of genomic DNA. Female *K14Cre.Ptpn2*^*fl/fl*^ and *K14CRE.Ptpn2*^*w/w*^ littermates at 7–8 weeks of age were used for the described experiments. The dorsal skin of each mouse was shaved 48 h before treatment; only those mice in the resting phase of hair cycle were used. All animal handling procedures were approved by the Institutional Animal Care and Use Committee at University of Texas Rio Grande Valley and were in accordance with the NIH Guide for the Care and Use of Laboratory Animals.

### Keratinocyte culture

3PC keratinocytes (an immortalized mouse keratinocytes cell line obtained from Ca^2+^-resistant adult keratinocytes after DMBA exposure)^52^ were obtained from University of Texas MD Anderson Cancer Center and cultured in low Ca^2+^ (0.03 mM) EMEM (Lonza, #06–174 G) containing 5 ng/ml EGF, 2.5 μg/ml insulin, 5 μg/ml hydrocortisone, 10 μg/ml transferrin, 10 μM ethanolamine, 10 μM phosphoethanolamine, 1% penicillin/streptomycin and 1% fetal bovine serum at 37 °C and 5% CO_2_ until 60 to 70% confluency at which time cells were treated with TPA. 3PC keratinocytes were verified by consistent morphological characteristic, with frozen aliquots from same passages (5 passages) used for experiments. Two day-old *K14Cre.Ptpn2*^*fl/fl*^ or *K14Cre.Ptpn2*^*w/w*^ neonates were used to culture primary keratinocytes as previously described^53^. Briefly, neonates were euthanized and skin was surgically removed. The skin was then placed in trypsin for 12 h at 4 °C to remove the dermis. Then, the isolated epidermis was minced and filtered by cell strainer (100 μm) to get rid of debris. Primary keratinocytes were plated and cultured at 37 °C and 5% CO_2_ in low Ca^2+^ (0.03 mM) KGM-2 medium (Lonza, #CC-3158) containing 1% penicillin/streptomycin and 1% fetal bovine serum until 60 to 70% confluency at which time cells were treated with DMBA or TPA.

### Knockdown of TC-PTP with siRNA and shRNA

3PC cells were grown overnight to ~40% confluence and transfected with ON-TARGET*plus*^TM^ mouse TC-PTP (*Ptpn2*) short interfering RNA (siRNA) and ON-TARGET*plus*^TM^ siCONTROL^®^ Non-Targeting pool siRNA as control (Thermo Scientific Dharmacon). Transfection was performed with Lipofectamine^®^ RNAiMAX (Invitrogen) according to the manufacturer’s instructions. TC-PTP-deficient stable cell lines established by lentiviral transduction with *Ptpn2*-specific shRNA were previously described^25^.

### Analysis of DMBA-induced apoptosis in primary keratinocytes and epidermis

Primary keratinocytes cultured from *K14Cre.Ptpn2*^*w/w*^ or *K14Cre.Ptpn2*^*fl/fl*^ neonates were treated with DMBA. Following incubation with DMBA for 24 h, keratinocytes presenting the morphological characteristics of apoptosis such as cell ballooning, nuclear condensation, and bleb formation were counted under phase contrast microscope. Apoptotic cells were counted from at least three non-overlapping fields. The percentage of apoptotic cells was calculated as follows: [number of apoptotic cells/(number of apoptotic cell plus number of viable cell)] × 100. To examine the effect of STAT3 or AKT on DMBA-induced apoptosis, keratinocytes were treated with STAT3 inhibitor S3I-201 (20 μM, Selleck Chemicals) or AKT1/2 inhibitor A6730 (20 μM, Sigma-Aldrich) 1 h before DMBA treatment. Capase-3 activity was measured by the caspase-3/CPP32 colorimetric assay kit (BioVision) according to the manufacturer’s instructions.

To analyze DMBA-induced epidermal apoptosis, groups of *K14Cre.Ptpn2*^*w/w*^ and *K14Cre.Ptpn2*^*fl/fl*^ mice (n = 3/group) were treated on the dorsal skin with a single topical application of DMBA (25 nmol) or acetone (200 μl) and sacrificed 24 h after treatment. Skin sections were stained with an antibody to the active form of caspase-3 (R&D systems) and then treated with biotinylated anti-rabbit IgG-conjugated ABD reagent (BD Biosciences). Again, apoptotic keratinocytes were counted manually under phase contrast from at least 3 random, non-overlapping fields of view of tissue sections from 3 individual mice.

### Flow cytometric analysis for apoptosis

Apoptosis of primary keratinocytes was evaluated using Annexin V-FITC apoptosis kit (Molecular probe, V13242) according of the manufacturer’s instructions. In brief, cells were harvested with 0.5% trypsin; 1 × 10^6^ cells were then washed cold PBS and centrifuged at 400 × g for 5 min and stained using Annexin V-FITC. Then, cell suspensions were analyzed using LSRFortessa (BD Biosciences). The apoptotic rate was calculated as Annexin V-positive cells. Each experiment was performed three times, and data were presented as means ± SD.

### Analysis of TPA-induced cell proliferation in primary keratinocytes

An equivalent number of primary keratinocytes obtained from *K14Cre.Ptpn2*^*fl/fl*^ or *K14Cre.Ptpn2*^*w/w*^ neonates were cultured in low calcium (0.03 mM) medium. After 48 h of culture, culture medium was changed to a medium containing TPA (40 nM) for 1 h. After 1 h pulse treatment, culture medium was replaced with low calcium medium for the remainder of the culture period. To determine the effect of STAT3 or AKT on keratinocyte proliferation, culture medium was replaced with either low calcium medium or low calcium medium containing one of the following: STAT3 inhibitor S3I-201 (10–50 μM); STAT3 inhibitor STA-21 (0.1–10 μM, Sigma-Aldrich); or AKT1/2 inhibitor A6730 (0.1–10 μM) before the 1 h pulse treatment with TPA. Cell proliferation was measured using a 2-(4-Iodophenyl)-3-(4-nitrophenyl)-5-(2,4-disulfophenyl)-2H-tetrazolium, monosodium salt (WST-1) (Dojindo Molecular Technologies) colorimetric assay.

### Analysis of epidermal thickness and cell proliferation following TPA treatment

For analysis of epidermal thickness and proliferation, groups of 7 weeks old, female *K14Cre.Ptpn2*^*w/w*^ and *K14Cre.Ptpn2*^*fl/fl*^ mice (n = 3/group) were treated with a single topical application of TPA (6.8 nmol) or acetone on the dorsal skin and sacrificed 24 and 48 h after treatment. Thirty minutes prior to sacrifice, mice were injected intraperitoneally with 5-bromo-2-deoxyuridine (BrdU) (Sigma-Aldrich) in PBS at 100 μg/g body weight. Dorsal skin was fixed in formalin and embedded in paraffin prior to sectioning at 4 μm and then histological slides were stained with hematoxylin and eosin. Epidermal thickness was measured from 30 interfollicular sites per each group. To measure TPA-induced epidermal cell proliferation, sections were stained with an anti-BrdU antibody (BD Biosciences, #3263611), followed by treatment with biotinylated anti-mouse IgG and horseradish peroxidase-conjugated ABC reagent (Vector Laboratories). Epidermal cell proliferation, presented as the labeling index, was quantified by calculating the percentage of basal cells positive for BrdU. A minimum of 500 basal cells was counted. For immunofluorescence analysis of proliferating cell nuclear antigen (PCNA), sections were stained with an anti-PCNA antibody (Abcam, #ab29), followed by incubation with secondary mouse Alexa Fluor^®^ 647 antibody (Invitrogen, #A21236). Sections were counterstained with 4,6-diamidino-2-phenylindole (DAPI) to highlight cell nuclei (Invitrogen, #R37606). The expression of PCNA in the epidermis was detected by the Leica DMI6000 B fluorescence microscope (Leica) at wavelengths of 405 and 633 nm.

### Two-stage skin carcinogenesis

Groups of 7 weeks old, female *K14Cre.Ptpn2*^*w/w*^ and *K14Cre.Ptpn2*^*fl/fl*^ littermates (n = 12/group) were initiated with 25 nmol of DMBA or acetone. Two weeks after initiation, mice received topical treatments with 6.8 nmol of TPA twice a week until the experiment was terminated. Tumor incidence (percentage of mice with papillomas) and tumor multiplicity (average number of papillomas per mouse) were determined weekly until multiplicity plateaued. Papillomas were measured at the end of the experiment by digital calipers and tumor surface was calculated. Carcinomas (average number of carcinomas per mouse) were also counted at the end of the experiment. Difference in tumor incidence and multiplicity were analyzed by the χ^2^ test and the Mann-Whitney *U* test, respectively.

### Preparation of protein lysates and western blot analysis

Total cell lysates were prepared from keratinocytes and epidermis with RIPA buffer (Thermo Fisher Scientific) containing 1% Triton X-100, protease inhibitor cocktail (Sigma-Aldrich) and phosphatase inhibitor cocktail I, II (Sigma-Aldrich). Equal amounts of total protein were resolved using SDS-PAGE and transferred to PVDF membrane (GE Healthcare). The membrane was incubated overnight at 4 °C with primary antibody, followed by incubation with secondary antibody conjugated to horseradish peroxidase. Enhanced chemiluminescence detection reagents (Amersham Biosciences) were used to detect immunoreactive protein. The following antibodies were used: anti-phospho-EGFR(#3777); anti-EGFR (#4627); anti-phospho-STAT3 (Tyr705) (#9145); anti-STAT3 (#9139); anti-phospho-AKT (#4060); anti-AKT (#4169); anti-phospho-Erk (#4370); anti-Erk (#4695); anti-Cycline D1(#2978); anti-Mcl-1 (#94296); anti-Bcl-xL (#2762); anti-Bax (#2772) (Cell Signaling Technology); anti-TC-PTP (R&D Systems, #MAB1930) and anti-β-actin (Sigma-Aldrich, #A5316).

### Immunohistochemical analysis

Formalin-fixed, paraffin-embedded tissues were deparaffinized and hydrated using standard procedures. Endogenous peroxidase activity was blocked with 0.03% hydrogen peroxide for 10 min. Sections were microwaved (10 min) in the presence of 10 mM citrate buffer (pH 6.0) containing 0.01% Tween 20 and allowed to cool for 20 min. Sections were then stained with an anti-TC-PTP (Proteintech, #11214-1-AP), p-STAT3 (#9145) and p-AKT (#4060) (Cell Signaling Technology) following suggested procedures by the manufacturer.

## Additional Information

**How to cite this article**: Lee, H. *et al*. Targeted disruption of TC-PTP in the proliferative compartment augments STAT3 and AKT signaling and skin tumor development. *Sci. Rep.* **7**, 45077; doi: 10.1038/srep45077 (2017).

**Publisher's note:** Springer Nature remains neutral with regard to jurisdictional claims in published maps and institutional affiliations.
